# PU.1 inhibition attenuates atrial fibrosis and atrial fibrillation vulnerability induced by angiotensin‐II by reducing TGF‐β1/Smads pathway activation

**DOI:** 10.1111/jcmm.16678

**Published:** 2021-06-15

**Authors:** Juan Hu, Jing‐Jing Zhang, Li Li, Shan‐Ling Wang, Hai‐Tao Yang, Xian‐Wei Fan, Lei‐Ming Zhang, Guang‐Ling Hu, Hai‐Xia Fu, Wei‐Feng Song, Li‐Jie Yan, Jing‐Jing Liu, Jin‐Tao Wu, Bin Kong

**Affiliations:** ^1^ Heart Center of Henan Provincial People’s Hospital Central China Fuwai Hospital Central China Fuwai Hospital of Zhengzhou University Zhengzhou China; ^2^ Department of Cardiology Renmin Hospital of Wuhan University Hubei China; ^3^ Department of Cardiology Qitai Farm Hospital Xinjiang China

**Keywords:** atrial fibrillation, atrial fibrosis, angiotensin‐II, PU.1

## Abstract

Fibrosis serves a critical role in driving atrial remodelling‐mediated atrial fibrillation (AF). Abnormal levels of the transcription factor PU.1, a key regulator of fibrosis, are associated with cardiac injury and dysfunction following acute viral myocarditis. However, the role of PU.1 in atrial fibrosis and vulnerability to AF remain unclear. Here, an in vivo atrial fibrosis model was developed by the continuous infusion of C57 mice with subcutaneous Ang‐II, while the in vitro model comprised atrial fibroblasts that were isolated and cultured. The expression of PU.1 was significantly up‐regulated in the Ang‐II‐induced group compared with the sham/control group in vivo and in vitro. Moreover, protein expression along the TGF‐β1/Smads pathway and the proliferation and differentiation of atrial fibroblasts induced by Ang‐II were significantly higher in the Ang‐II‐induced group than in the sham/control group. These effects were attenuated by exposure to DB1976, a PU.1 inhibitor, both in vivo and in vitro. Importantly, in vitro treatment with small interfering RNA against Smad3 (key protein of TGF‐β1/Smads signalling pathway) diminished these Ang‐II‐mediated effects, and the si‐Smad3‐mediated effects were, in turn, antagonized by the addition of a PU.1‐overexpression adenoviral vector. Finally, PU.1 inhibition reduced the atrial fibrosis induced by Ang‐II and attenuated vulnerability to AF, at least in part through the TGF‐β1/Smads pathway. Overall, the study implicates PU.1 as a potential therapeutic target to inhibit Ang‐II‐induced atrial fibrosis and vulnerability to AF.

## 1 INTRODUCTION

Atrial fibrillation (AF) can result in heart failure, stroke and increased cardiac mortality and morbidity.^1^, ^2^ Atrial remodelling serves an important role in the pathological mechanism of AF, and atrial fibrosis is the crucial substrate for atrial remodelling.^3^, ^4^, ^5^ Therefore, attenuating atrial fibrosis is considered an effective strategy for preventing the occurrence and maintenance of AF.

Transforming growth factor‐β1 (TGF‐β1), an important fibrogenic factor, binds its type II receptor (TβRII) and type I receptor (TβRI) to activate a cascade of phosphorylation reactions that promote inactivation of the Smad proteins 2, 3 and 4, before finally translocating to the nuclei and regulating the expression of profibrotic proteins, including connective tissue growth factor (CTGF) and collagen.^1^, ^6^ There is substantial evidence that angiotensin‐II (Ang‐II) can up‐regulate TGF‐β1 and collagen expression in vitro and in vivo, and Ang‐II plays an important role in atrial fibrosis and AF through its regulation of TGF‐β1.^7^, ^8^, ^9^, ^10^, ^11^ Therefore, inactivation of the TGF‐β1/Smads pathway attenuates the atrial fibrosis induced by Ang‐II.

PU.1, a member of the ETS family of transcription factors, is a vital regulator of gene expression, particularly in macrophages, B cells, T cells and dendritic cells.^12^, ^13^ PU.1 binds its recognition motifs to facilitate DNA methyltransferase activity, chromatin opening and the binding of other transcription initiation factors.^14^, ^15^ PU.1 is also a reprogramming factor that converts fibroblasts or neural stem cells into monocytes, macrophages and dendritic cells,^16^, ^17^, ^18^, ^19^, ^20^ and deregulation of PU. 1 is considered a crucial contributor to leukaemia pathogenesis.^21^, ^22^ Research has revealed that PU.1 is up‐regulated in the fibroblasts in various fibrotic diseases.^23^, ^24^, ^25^, ^26^, ^27^, ^28^ A recent report has found that the majority of PU.1^+^ cells in fibrotic tissues are fibroblasts; in contrast, PU.1^+^ fibroblasts are not found in normal or inflamed tissues of the skin, lung, liver, kidney or joints, as TGF‐β1 promotes PU.1 expression in fibrotic fibroblasts but not in resting fibroblasts or inflammatory fibroblasts; interestingly, Smad3 inhibition significantly reduces the PU.1 expression induced by TGF‐β1 in fibrotic fibroblasts.^26^ These findings support the hypothesis that PU.1 can control the fibroblast polarization and tissue fibrosis mediated by the TGF‐β1/Smads pathway. However, whether PU.1 regulates Ang‐II‐induced atrial fibrosis via the TGF‐β1/Smads pathway remains unclear. Based on these observations, we developed a mouse model of atrial fibrosis induced by continuous subcutaneous Ang‐II infusion to reveal whether PU.1 inhibition attenuates Ang‐II‐induced atrial fibrosis and vulnerability to AF via the TGF‐β1/Smads pathway.

## 2 MATERIALS AND METHODS

### 2.1 Animals

All animal experiments were approved by the Animal Care and Use Committee of Renmin Hospital at Wuhan University and performed in accordance with the Guide for the Care and Use of Laboratory Animals published by the US National Institutes of Health (8th Edition, NRC 2011). Male C57BL/6 mice were housed under standard conditions with a controlled temperature, humidity and 12‐h light/dark cycle. Food and water were provided ad libitum. An osmotic minipump (Model 1002; Alzet, Cupertino, CA, USA) was implanted in the mice for the subcutaneous infusion of Ang‐II (750 ng/kg/min) or phosphate‐buffered saline (sham) for 28 days.^8^ The mice were divided into four groups: sham (no Ang‐II induction), sham+DB1976 (a PU.1 inhibitor), Ang‐II and Ang‐II+DB1976. Mice in the sham and Ang‐II groups were given saline, while mice in the sham+DB1976 group and Ang‐II+DB1976 group were administered with DB1976 (5 mg/day/kg; Glpbio, Montclair, CA, USA) by intraperitoneal injection once a day for 28 days, as previously described.^26^ Mice were anaesthetized with 50 mg/kg sodium pentobarbital (Merck KGaA, Darmstadt, Germany) prior to the assays.

### 2.2 Quantitative real‐time PCR (qRT‐PCR)

RNA was purified from the atrium samples using TRIzol reagent (Thermo Fisher Scientific, Waltham, MA, USA) and transcribed into cDNA with the PrimeScript RT Reagent Kit (Takara Bio, Shiga, Japan). Next, qRT‐PCR was conducted in a 20‐µL reaction system containing cDNA, forward and reverse primers, and the SYBR Premix Ex Taq (Takara). GAPDH was used as the internal control. The sequences of the PU.1 primers were as follows: forward, 5′‐CAGGGATGATGTTCTGGGCA‐3′; reverse, 5′‐ACTCTGCAGCTCTGTGAAGT‐3′. The sequences of the GAPDH primers were as follows: forward, 5′‐ATGGGTGTGAACCACGAGA‐3′; reverse, 5′‐CAGGGATGATGTTCTGGGCA‐3′.

### 2.3 Western blot analysis

Proteins were extracted from the frozen atrium tissues, and the protein concentrations were determined using the Bicinchoninic Acid Protein Assay Kit (Abcam, Cambridge, UK) according to previously described.^29^ Proteins (40 mg per lane) were separated by sodium dodecyl sulphate‐polyacrylamide gel electrophoresis (SDS‐PAGE), transferred onto a polyvinylidene difluoride (PVDF) membrane and incubated with the indicated primary antibodies overnight at 4℃. The primary antibodies included anti‐PU.1 (1:1000; Abcam), anti‐TGF‐β1 (1:1000; Abcam), anti‐p‐Smad3 (1:1000; Cell Signaling Technology, Danvers, MA, USA), anti‐p‐Smad2/3 (1:1000; Cell Signaling Technology), anti‐α‐SMA (1:1000; Cell Signaling Technology), anti‐PCNA (1 μg/mL, Abcam), anti‐collagen I (1:1000; Abcam), anti‐CTGF (1:1000; Abcam), anti‐Discoidin domain receptor 2 (DDR2) (1:1000; Abcam), anti‐ED‐A fibronectin (ED‐A Fn) (1:1000; Millipore, Bedford, MA, USA), anti‐embryonic smooth muscle myosin heavy chain (SMemb) (1:1000; Abcam) and anti‐GAPDH (1:1000; Abcam). Finally, the membranes were incubated with the secondary horseradish peroxidase (HRP)‐goat anti‐rabbit (AS1107; 1:10 000; Aspen Biotechnology, Bedford, MA) or HRP‐goat anti‐mouse (AS1106; 1:10 000; Aspen Biotechnology) at room temperature for 1 hr and exposed to enhanced chemiluminescence (ECL) substrate (AS1027; Aspen Biotechnology). Western blot results were quantified by densitometry (Image Lab, Hercules, CA). The relative intensities of the proteins of interest were normalized to that of the sham/control group, which was set to a value of 1 (100%).

### 2.4 Histological studies

The heart of each mouse was removed, fixed in 4% paraformaldehyde and embedded in paraffin. Atrium tissue sections (4 μm thick) were stained with Masson's trichrome, visualized with an Olympus BX51 microscope (Olympus Corporation, Tokyo, Japan) and analysed using Image‐Pro Plus 6.0 software (Media Cybernetics, Inc, Rockville, MD, USA). The percentage of fibrosis was measured by calculating the ratio of fibrotic tissue area to that of the normal myocardial tissue.

### 2.5 Adenoviral vector construction

For the overexpression of PU.1 in mice, a replication‐defective adenoviral vector was used that contained the entire coding region of the PU.1 gene (*Spi1*) under the control of the cytomegalovirus promoter, which we termed Ad‐PU.1 (JiKai, Shanghai, China). An adenoviral vector encoding GFP (Ad‐GFP) was used as the control. Atrial fibroblasts were transfected with Ad‐GFP or Ad‐PU.1 at a multiplicity of infection of 50, and no cytotoxic effects were detectable in 95% to 100% of the transfected cells (data not shown).

### 2.6 Isolation, culture and treatment of atrial fibroblasts

Atrial fibroblasts from the Ang‐II‐infused mice were isolated as previously described^8^ and cultured in high‐glucose Dulbecco's modified Eagle medium (DMEM) supplemented with 10% foetal bovine serum (FBS) and 1% penicillin/streptomycin. All cells from the first or second passages were serum‐starved for 24 h before the downstream experiments. Atrial fibroblasts were transfected with negative control (nonsense) small interfering RNA (con‐siRNA; 100 nM), a siRNA against Smad3 (siRNA‐Smad3; 100 nM) or a siRNA against PU.1 (siRNA‐PU.1; 100 nM) using Lipofectamine 2000 (Thermo Fisher Scientific) according to the manufacturer's instructions. For the PU.1 inhibition assay, the cells were exposed to DB1976 (2.5 μM) or siRNA‐PU.1(100 nM) for 24 h before collecting the cells. For experiments analysing the effect of PU.1 overexpression, the cells were transfected with the Ad‐PU.1 or Ad‐GFP (control) adenoviral vector at a multiplicity of infection of 100 for 6h and cultured in fresh medium for a further 48 h and then treated with or without Ang‐II (1 *u*M) or si‐Smad3 (100 nM) for 24 h and finally collected the cells for assays.

### 2.7 Atrial fibroblast proliferation assay

The proliferation of atrial fibroblasts isolated from Ang‐II‐infused mice was measured with the Cell Counting Kit‐8 (CCK‐8; Dojindo Molecular Technologies, Kumamoto, Japan) according to the manufacturer's instructions.^30^ Atrial fibroblasts were plated in 96‐well plates, serum‐starved for 24 h and exposed to Ang‐II, DB1976, siRNA‐Smad3 or Ad‐PU.1 according to our assays. Following incubation, CCK‐8 solution was added for 4 h and the absorbance at 450 nm (OD_450_) was read with a Sunrise microplate reader (Tecan Group, Ltd., Männedorf, Switzerland).

### 2.8 Immunofluorescence

The atrium tissue samples were fixed and sectioned for analysis as previously described.^31^ The atrium tissue sections were incubated with anti‐PU.1 (1:100; Abcam), anti‐PCNA (5 μg/mL; Abcam) and anti‐α‐SMA (1:50; Cell Signaling Technology) primary antibodies at 4℃ overnight, followed by incubation with a fluorescein isothiocyanate (FITC)–conjugated secondary antibody (1:1000; Cell Signaling Technology) and visualization with an Olympus BX51 microscope (Olympus Corporation).

For immunofluorescence assays of the atrial fibroblasts, the cells were washed with phosphate‐buffered saline (PBS) and fixed with 4% paraformaldehyde for 20 min, followed by permeabilization with 0.1% Triton X‐100 in PBS. The cells were then added to coverslips with a syringe, and the coverslips were blocked with 3% bovine serum albumin for 30 min and then incubated overnight with primary antibodies against PU.1 (1:100; Abcam), anti‐PCNA (5 μg/mL; Abcam) and anti‐α‐SMA (1:50; Cell Signaling Technology). The next day, the coverslips were incubated with a FITC‐conjugated secondary antibody for 1 h, and the nuclei were stained with 4′,6‐diamidino‐2‐phenylindole (DAPI) for 30 min. Cells were then imaged with an Olympus BX51 microscope.

### 2.9 Electrophysiological studies using isolated perfused hearts

Langendorff‐perfused hearts were isolated as previously described.^32^ Electrophysiological studies on the perfused hearts were carried out using the Langendorff apparatus (AD Instruments, Bella Vista, Australia) with 4‐(2‐hydroxyethyl)‐1‐piperazineethanesulfonic acid (HEPES)–buffered Tyrode's solution (10 mM HEPES, pH 7.4, 130 mM NaCl, 1.8 mM CaCl_2_, 5.4 mM KCl, 1 mM MgCl_2_, 0.3 mM Na_2_HPO_4_, 10 mM glucose) bubbled through with 95% O_2_ and 5% CO_2_ at 37℃ at a constant pressure of 60 mmHg to evaluate the induction of AF as previously described.^33^ Each heart was perfused until stabilization for 20 min before conducting the programmed electrophysiology tests. Hearts that exhibited irreversible myocardial ischaemia or did not recover to a regular spontaneous rhythm were discarded.^34^ Teflon‐coated (except at the tips) silver bipolar electrodes were placed on the appendages of the right atrium and left atrium. AF inducibility was tested with the burst pacing method, as previously described.^8^ AF was defined as a rapid irregular atrial rhythm with irregular RR intervals lasting for at least 5 s. The duration of AF was measured from the end of burst pacing to the first P wave detected after the rapid irregular atrial rhythm.

### 2.10 Statistical analysis

All data are presented as the mean ±standard deviation (SD). Student's *t* test was used to determine differences between two groups, and one‐way analysis of variance (ANOVA) was used for multiple group comparisons. All data analysis was performed with SPSS Statistics software v. 20.0 (IBM Corp., Armonk, NY, USA). A *P* value of <0.05 was considered statistically significant.

## 3 RESULTS

### 3.1 PU.1 expression in atrial tissue and atrial fibroblasts is induced by Ang‐II in vivo and in vitro

**FIGURE 1:**
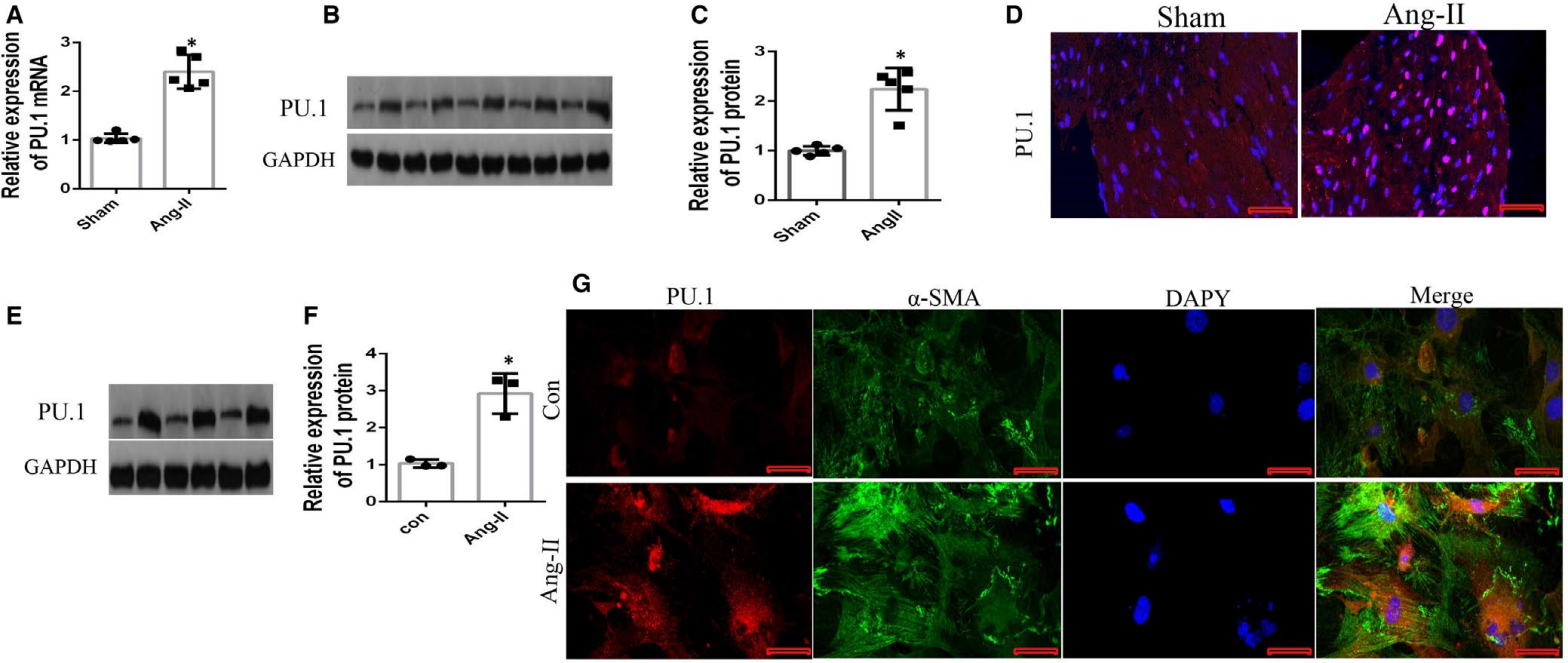
PU.1 expression in mice atrial tissue or fibroblasts was induced by Ang‐II in vivo and in vitro, respectively. The expression of PU.1 was evaluated by qRT‐PCR, Western blotting and immunofluorescence assays of atrial tissue following induction by subcutaneous infusion with Ang‐II for 28 days and in cultured atrial fibroblasts sourced from the Ang‐II‐induced mice with or without Ang‐II for 24 hours. (A) mRNA expression of the gene encoding PU.1 (n = 5). (B) Representative Western blots and (C) quantitative densitometric analyses of PU.1 expression in vivo (n = 5). (D) Representative photomicrographs of PU.1 expression in vivo. Red staining indicates PU.1 expression. Nuclei were counterstained with DAPI. Scale bar, 20 µm (n = 5). (E) Representative Western blots and (F) quantitative densitometric analyses of PU.1 expression in vitro (n = 3). (G) Representative photomicrographs of PU.1 expression in vitro (n = 3). Red staining indicates PU.1 expression, and green staining indicates α‐SMA expression. Nuclei were counterstained with DAPI. Scale bar, 20 µm. Data represent the mean ± SD. *P < .01 vs. the sham/control group. GAPDH was used as the internal control. Ang‐II, angiotensin‐II; DAPI, 4′,6‐diamidino‐2‐phenylindole

To assess the effect of PU.1 on Ang‐II‐mediated fibrosis, PU.1 expression following induction by Ang‐II was assessed in atrial tissue and atrial fibroblasts in vivo and in vitro, respectively. qRT‐PCR, Western blotting and immunofluorescence staining analyses demonstrated that PU.1 expression was significantly higher (*P* < .01) in the Ang‐II‐induced group compared with the sham group in vivo (Figure 1A–D). Next, we isolated atrial fibroblasts from mice subjected to the subcutaneous infusion of Ang‐II and performed Western blot and immunofluorescence analyses of PU.1 expression. PU.1 expression was significantly greater (*P* < .01) in the Ang‐II‐stimulated group compared with the control group (Figure 1E–F). Immunofluorescence staining indicated that double‐positive cells of PU.1 and α‐SMA (specific marker of myofibroblasts) expression were also increased in the induced atrial fibroblasts (Figure 1G). These findings suggest that PU.1 is associated with Ang‐II‐mediated atrial fibrosis.

### 3.2 Inhibition of PU.1 down‐regulated activation of the atrial TGF‐β1/Smads pathway induced by Ang‐II in vivo and in vitro

**FIGURE 2:**
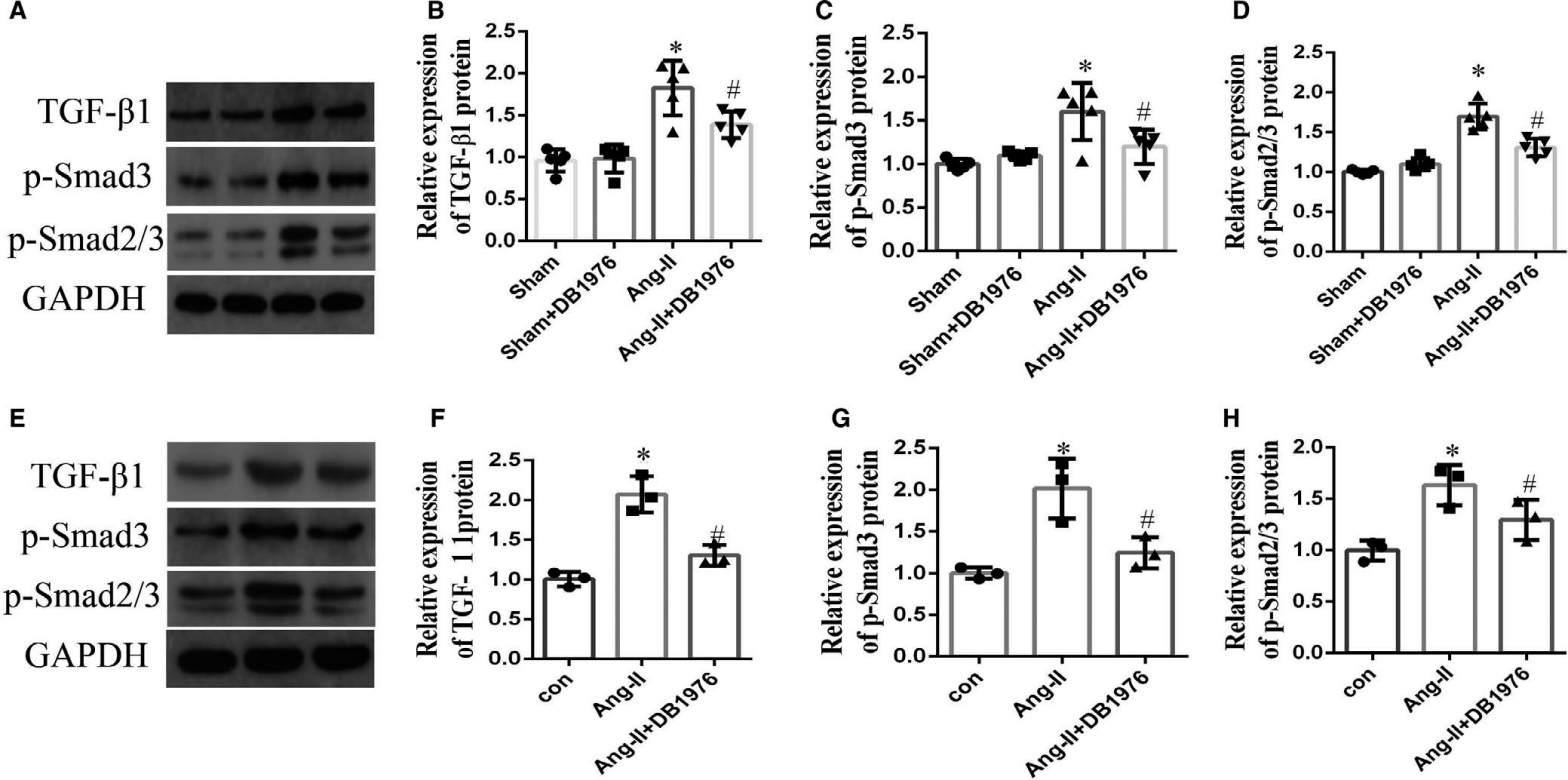
Inhibition of PU.1 in mice down‐regulated the Ang‐II‐induced activation of the atrial tissue and atrial fibroblasts TGF‐β1/Smads pathway in vivo and in vitro. (A) Representative Western blots and (B–D) quantitative densitometric analyses showing the expression of TGF‐β1, p‐Smad3 and p‐Smad2/3 in the atrial tissue induced by the subcutaneous infusion of Ang‐II with or without DB1976 (n = 5). (E) Representative Western blots and (F–H) quantitative densitometric analyses showing the expression of TGF‐β1, p‐Smad3 and p‐Smad2/3 in the cultured atrial fibroblasts sourced from Ang‐II‐induced mice and treated with and/or Ang‐II/DB1976 for 24 h (n = 3). Data represent the mean ± SD. *P < .01 vs. the sham/control group, ^#^ *P* < .05 vs. the Ang‐II group. GAPDH was used as the internal control. Ang‐II, angiotensin‐II

The Smads signalling pathway plays a key role in regulating tissue fibrosis, and Ang‐II is considered to induce the synthesis and release of fibrosis factors via the TGF‐β1/Smads pathway.^11^ A recent report suggested that PU.1 promotes skin, lung, liver, joint and kidney fibrosis by regulating the activity of the TGF‐β1/Smad3 pathway.^26^ However, whether PU.1 participates in the atrial fibrosis induced by Ang‐II via the TGF‐β1/Smads pathway is unknown. Therefore, in this study, we aimed to assess whether PU.1 influences the atrial fibrosis induced by Ang‐II via the TGF‐β1/Smads pathway. Western blot analyses revealed that the TGF‐β1, p‐Smad3 and p‐Smad2/3 protein levels were significantly up‐regulated (*P* < .01) in the Ang‐II‐induced group compared with the sham, and this up‐regulation was partially blocked in vivo by exposure to the PU.1 inhibitor, DB1976 (Figure 2A–D, *P* < .05). Moreover, similar effects were found in vitro (Figure 2E–H, P <.05). Overall, these results indicate that PU.1 is involved in the pathological processes induced by Ang‐II via the TGF‐β1/Smads pathway.

### 3.3 Inhibition of PU.1‐attenuated Ang‐II‐induced atrial fibroblast proliferation and differentiation in vivo and in vitro

**FIGURE 3:**
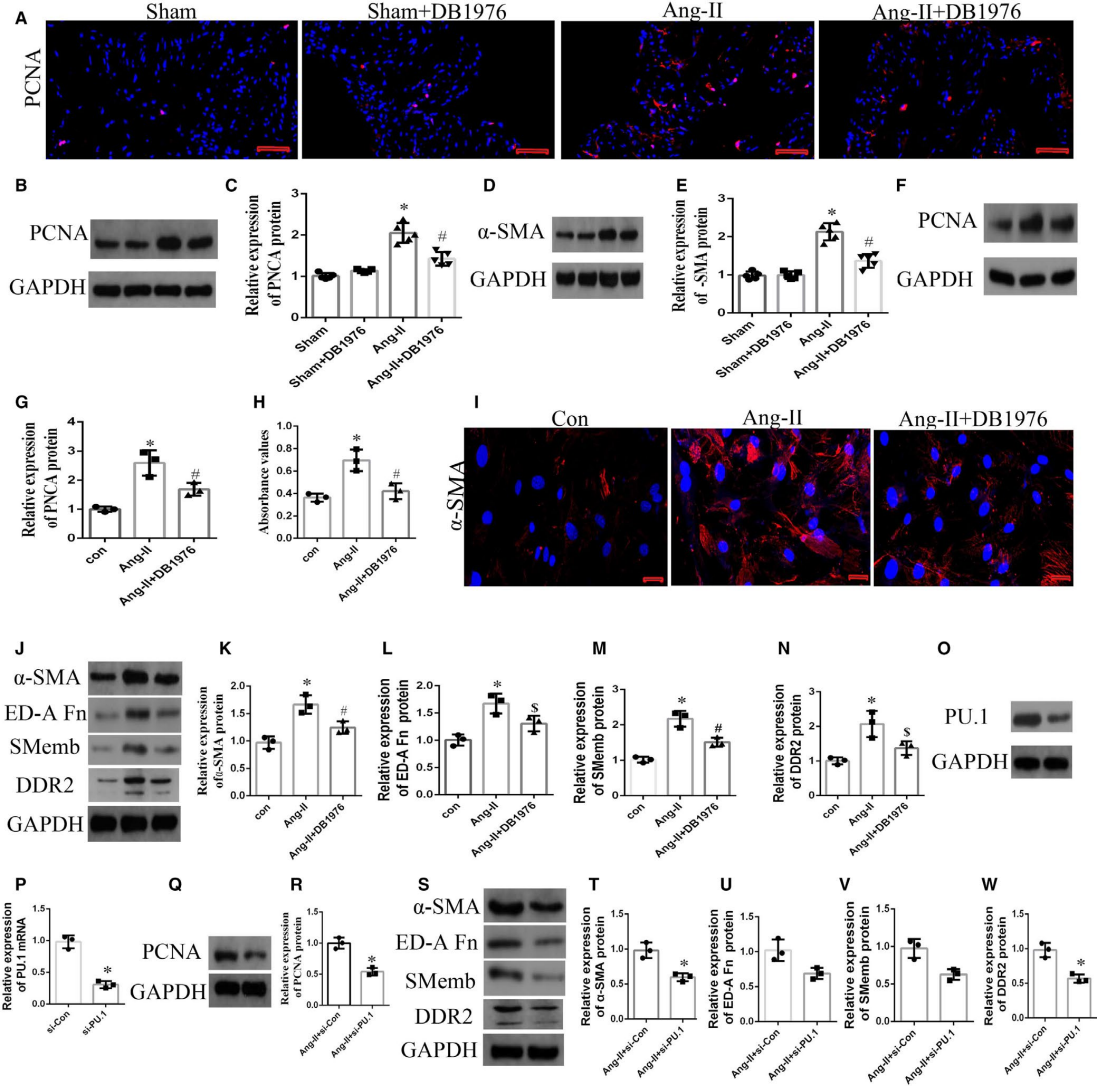
Inhibition of PU.1 in mice attenuated the atrial fibroblast proliferation and differentiation induced by Ang‐II in vivo and in vitro. (A) Representative immunofluorescence images of PCNA expression in the atrial tissue of mice induced by the subcutaneous infusion of Ang‐II. Red staining indicates PCNA expression. Nuclei were counterstained with DAPI. Scale bar, 20 µm. (B, D) Representative Western blots and (C, E) quantitative densitometric analyses showing the expression of PCNA and α‐SMA in the atrial tissue inducted by Ang‐II in vivo (n = 5). (F‐N) Atrial fibroblasts sourced from Ang‐II‐induced mice treated with and/or Ang‐II/DB1976 for 24 h. (F, J) Representative Western blots and (G, K, L, M, N) quantitative densitometric analyses showing the expression of PCNA, α‐SMA, ED‐A Fn, Smemb and DDR2; (H) Absorbance values of the CCK‐8 proliferation assay; (I) representative immunofluorescence images of α‐SMA expression (red staining). Scale bar, 20 µm. (O‐W) Atrial fibroblasts sourced from Ang‐II‐induced mice treated with and/or Ang‐II/si‐PU.1 for 24 h. (O, Q, S) Representative Western blots and (P, R, T, U, V, W) quantitative densitometric analyses showing the expression of PU.1, PCNA, PCNA, α‐SMA, ED‐A Fn, Smemb and DDR2 (n = 3). Data represent the mean ± SD. *P < .01 vs. the sham/control group. ^#^ *P* < .05 vs. the Ang‐II group/Ang‐II + si‐Con group. GAPDH was used as the internal control. Ang‐II, angiotensin‐II; CCK‐8, Cell Counting Kit‐8; DAPI, 4′,6‐diamidino‐2‐phenylindole

To determine the effect of PU.1 on atrial fibroblast proliferation and differentiation in atrial tissue isolated from mice induced with Ang‐II, double immunofluorescence staining was performed for proliferating cell nuclear antigen (PCNA). As shown in Figure 3A, the number of PCNA‐positive cells was lower in the Ang‐II+DB1976 group compared with the Ang‐II group by immunofluorescence staining in vivo (Figure 3A). Moreover, Western blotting indicated that the expression of PCNA and a‐SMA was significantly higher (*P* < .01) in the Ang‐II‐induced group than in the sham group, and these increases were attenuated by PU.1 inhibition in the Ang‐II + DB1976 group (Figure 3B–E, *P* < .05). Next, cultured atrial fibroblasts isolated from Ang‐II‐induced mice were subjected to Western blotting, immunofluorescence staining and CCK‐8 assays to assess cell proliferation and differentiation. Especially, α‐SMA, SMemb and ED‐A Fn are main markers of myofibroblast^35^; in addition, DDR2 determines a‐SMA‐dependent collagen gene expression in the Ang‐II‐induced cardiac fibroblasts.^36^ Therefore, we measured the above indicators, and the results further validated the in vivo findings (Figure 3F‐N). To further confirm the above effects, PU.1 was knocked down in the cultured atrial fibroblasts using siRNA. The results showed that the levels of PU.1, PCNA, α‐SMA, SMemb, ED‐A Fn and DDR2 were significantly decreased (*P* < .05) in Ang‐II‐induced atrial fibroblasts transfected with si‐PU.1 (Figure 3O‐W). These findings suggest that PU.1 inhibition attenuates Ang‐II‐induced atrial fibroblast proliferation and differentiation.

### 3.4 Inhibition of PU.1‐attenuated Ang‐II‐induced atrial fibrosis in vivo and in vitro

**FIGURE 4:**
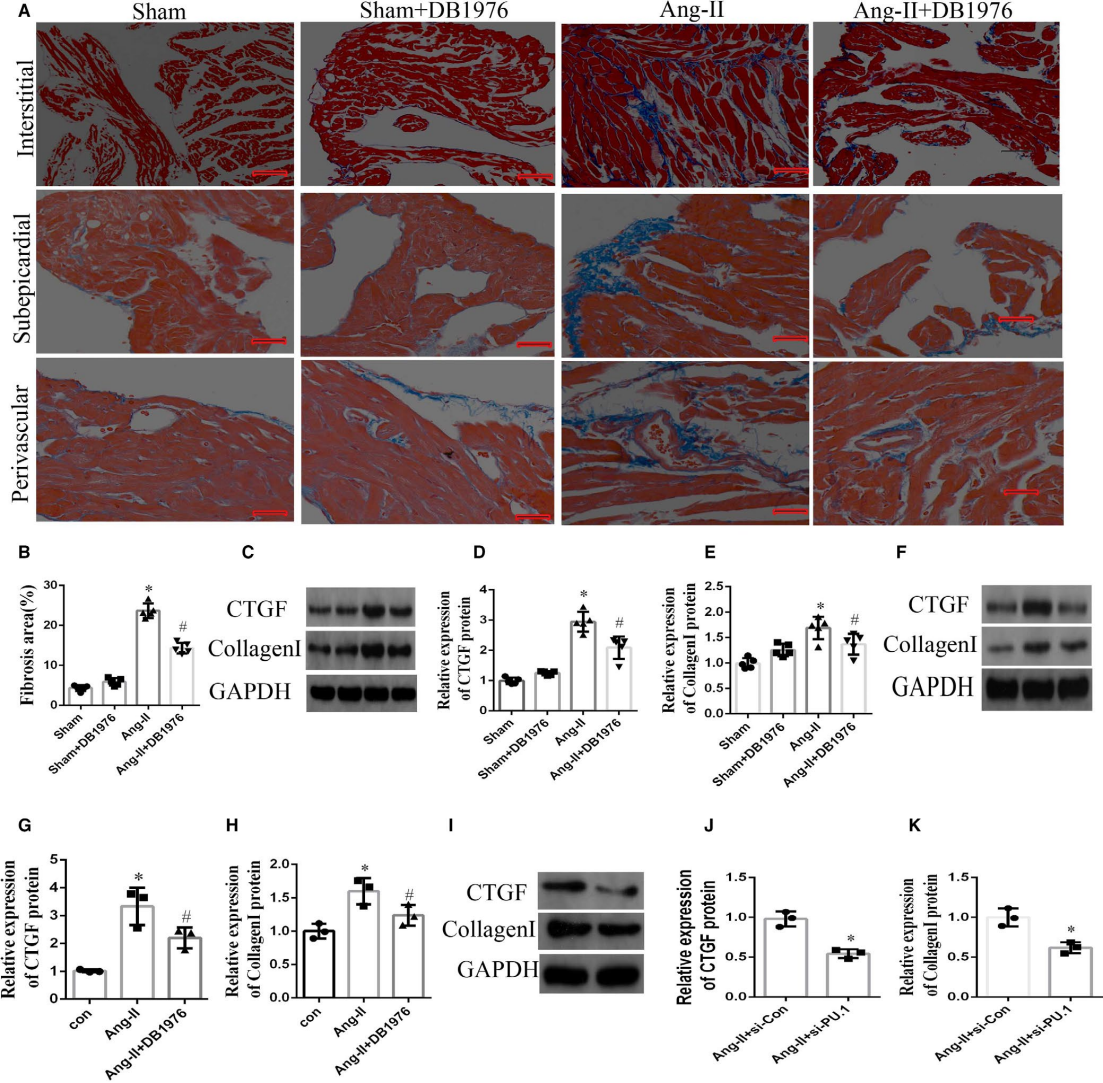
Inhibition of PU.1 in mice attenuated the atrial fibrosis induced by Ang‐II in vivo and in vitro. (A) Representative images of Masson's trichrome staining of the interstitial, sub‐epicardial and perivascular regions of the atrial tissue. Blue staining indicates the fibrotic tissue. Scale bar, 20 µm. (B) Quantitative analysis of fibrosis (n = 5). (C) Representative Western blot and (D, E) quantitative densitometric analyses showing the expression of CTGF and collagen I in the atrial tissue induced by the subcutaneous infusion of Ang‐II (n = 5). (F, I) Representative Western blots and (G, H, J, K) quantitative densitometric analyses showing the expression of CTGF and collagen I in the cultured atrial fibroblasts sourced from the Ang‐II‐induced mice and treated with or without Ang‐II, DB1976 or si‐PU.1 for 24 h (n = 3). Data represent the mean ± SD. *P < .01 vs. the sham/control group, ^#^ *P* < .05 vs. the Ang‐II group/Ang‐II + si‐Con group. GAPDH was used as the internal control. Ang‐II, Angiotensin‐II; si‐PU.1, small interfering RNA against PU.1

To examine the effects of PU.1 on Ang‐II‐induced atrial fibrosis, Ang‐II‐induced mice and cultured atrial fibroblasts sourced from these mice were exposed to DB1976. Masson's trichome staining of the atrial tissue showed that DB1976 exposure significantly decreased (*P* < .05) the interstitial, sub‐epicardial and perivascular fibrosis induced by Ang‐II in the atrial tissue (Figure 4A, B), and Western blotting indicated that fibrotic markers in the atrial tissue (CTGF and collagen I) were significantly more prominent (*P* < .01) in the Ang‐II‐induced group than in the sham group. Moreover, exposure to DB1976 reduced the fibrotic effects of Ang‐II (Figure 4C–E, P <.05).

To further confirm the effect of PU.1 on Ang‐II‐induced atrial fibrosis, atrial fibroblasts were isolated from Ang‐II‐induced mice and cultured. The levels of CTGF and collagen I were significantly higher (*P* <.01) in the Ang‐II‐induced group compared with the control group; these increases in expression were reversed by DB1976 treatment (Figure 3F–H; *P* < .05). Further, transfection of the atrial fibroblasts with si‐PU.1 significantly decreased (*P* < .05) the increased protein expression levels of CTGF and collagen I induced by Ang‐II (Figure 3I–K). These results reveal that PU.1 likely plays a key role in the pathological process of Ang‐II‐induced atrial fibrosis.

### 3.5 Inhibition of PU.1‐blunted electrical remodelling of the atrium in Ang‐II‐induced mice

**FIGURE 5:**
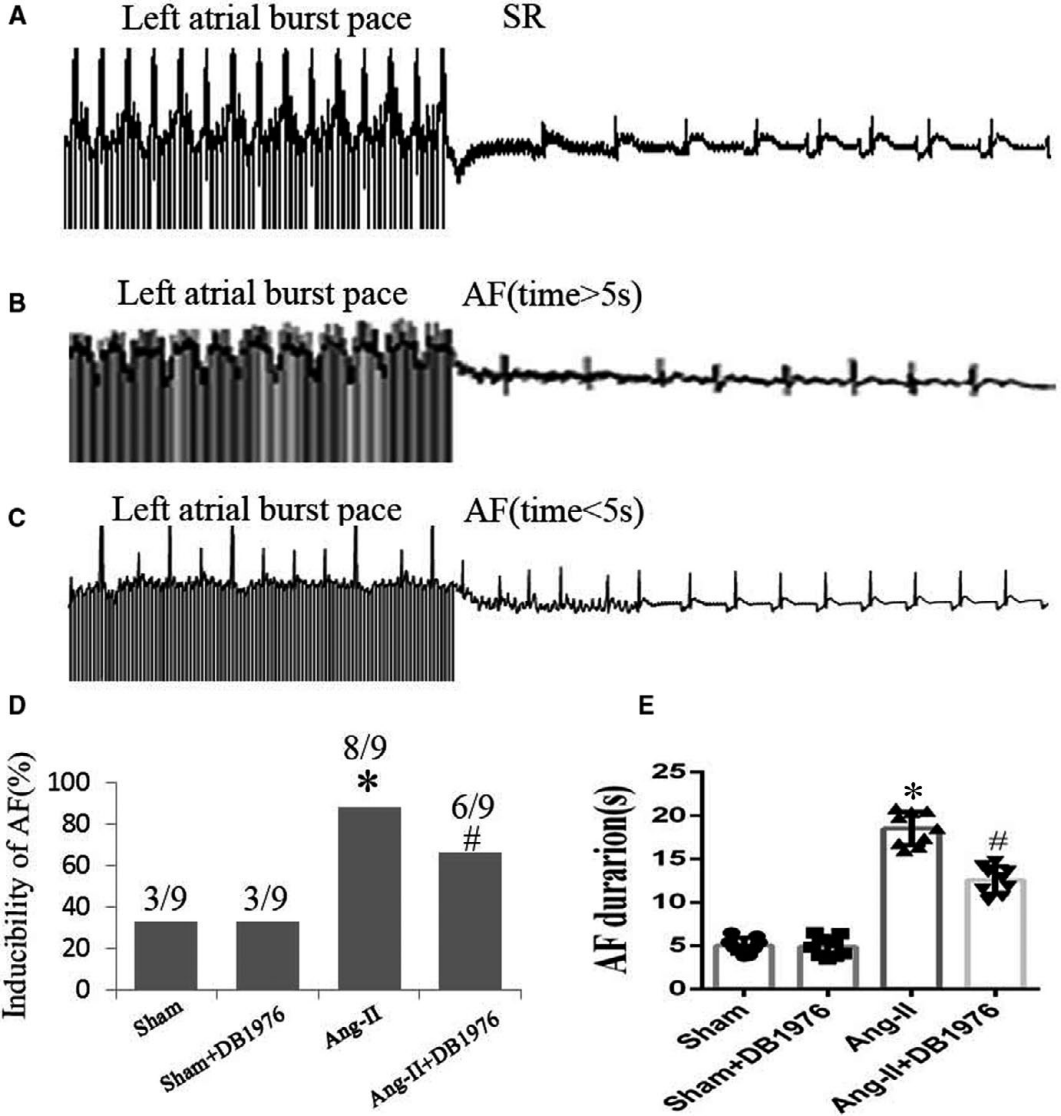
Inhibition of PU.1 in mice blunted electrical remodelling of the atrium induced by Ang‐II. Langendorff perfusion was performed on hearts prepared from mice in the four treatment groups indicated. (A‐C) Representative normal and AF responses induced by burst pacing stimulations. (D‐E) Summary of the AF inducibility rates and AF duration (n = 9). Data represent the mean ± SD. *P < .01 vs. the sham group, ^#^ *P* <.05 vs. the Ang‐II group. AF, atrial fibrillation; Ang‐II, angiotensin‐II

Based on the observation that PU.1 inhibition attenuated Ang‐II‐induced atrial fibrosis, we hypothesized that PU.1 modulates cardiac electrophysiological properties. Therefore, we performed an in vivo electrophysiology study of Langendorff‐perfused hearts using a method previously described.^32^, ^33^ The resulting data revealed that vulnerability to AF and AF duration were significantly higher (*P* < .01) in the Ang‐II‐induced group than in the sham group, whereas these parameters in the DB1976‐exposed group were significantly lower (*P* < .05) than in the Ang‐II group (Figure 5A–E). These findings indicate that PU.1 inhibition in vivo decreases Ang‐II‐induced AF vulnerability and AF duration by at least in part through decreasing atrial fibrosis.

### 3.6 Effects of PU.1 overexpression on Ang‐II‐induced atrial fibroblast proliferation, differentiation and fibrosis are dependent on the TGF‐β1/Smads pathway

**FIGURE 6:**
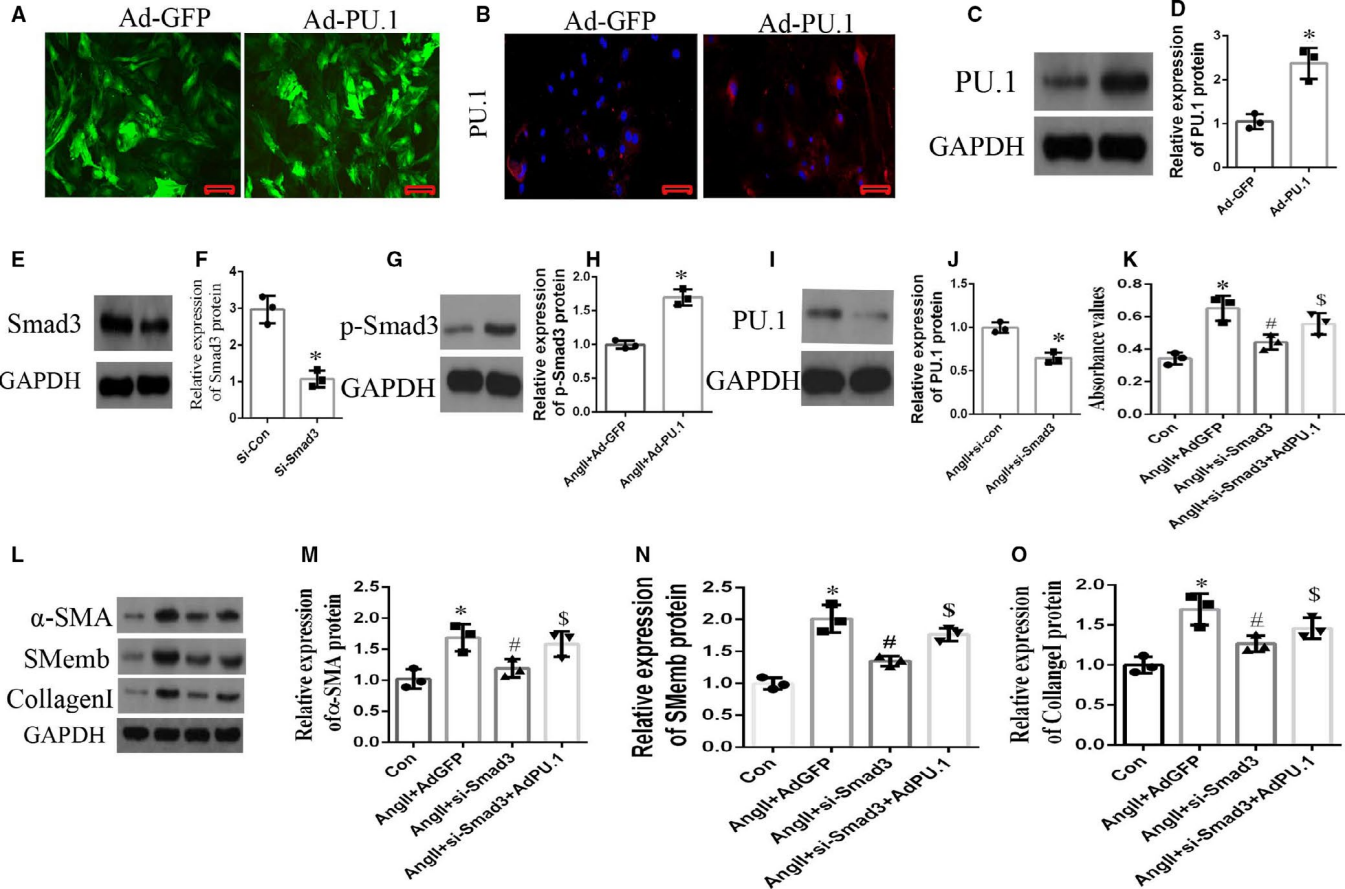
Effects of PU.1 on Ang‐II‐induced atrial fibroblast proliferation, differentiation and fibrosis are dependent on the TGF‐β1/Smads pathway. The cultured atrial fibroblasts sourced from Ang‐II‐induced mice were transfected with the Ad‐GFP or Ad‐PU.1 adenoviral vector for 6 h, cultured in fresh medium for a further 48 h and then treated with or without Ang‐II or si‐Smad3 for 24 h. (A) Representative photomicrographs of the induced atrial fibroblasts transfected with Ad‐GFP or Ad‐PU.1 for 48 h. (B) Representative immunofluorescence images of PU.1 expression in induced atrial fibroblasts treated with Ad‐GFP or Ad‐PU.1 for 48 h. Red staining indicates PU.1 expression. Nuclei were counterstained with DAPI. Scale bar, 50 µm. (C, E, G, I, L) Representative Western blots and (D, F, H, J, M, N, O) quantitative densitometric analyses of the relative PU.1, Smad3, p‐Smad3, α‐SMA, SMemb and collagen I protein expression normalized to GAPDH levels. (K) CCK‐8 proliferation assay absorbance values. Data represent the mean ± SD. *P < .01 vs. the Ad‐GFP/si‐con/Ang‐II + Ad‐GFP/Ang‐II + si‐con/Con group, ^#^ *P* < .05 vs. the Ang‐II + Ad‐GFP group. ^$^ *P* < .05 vs. the Ang‐II + si‐Smad3 group. Ang‐II, angiotensin‐II; CCK‐8, Cell Counting Kit‐8; DAPI, 4′,6‐diamidino‐2‐phenylindole

**FIGURE 7:**
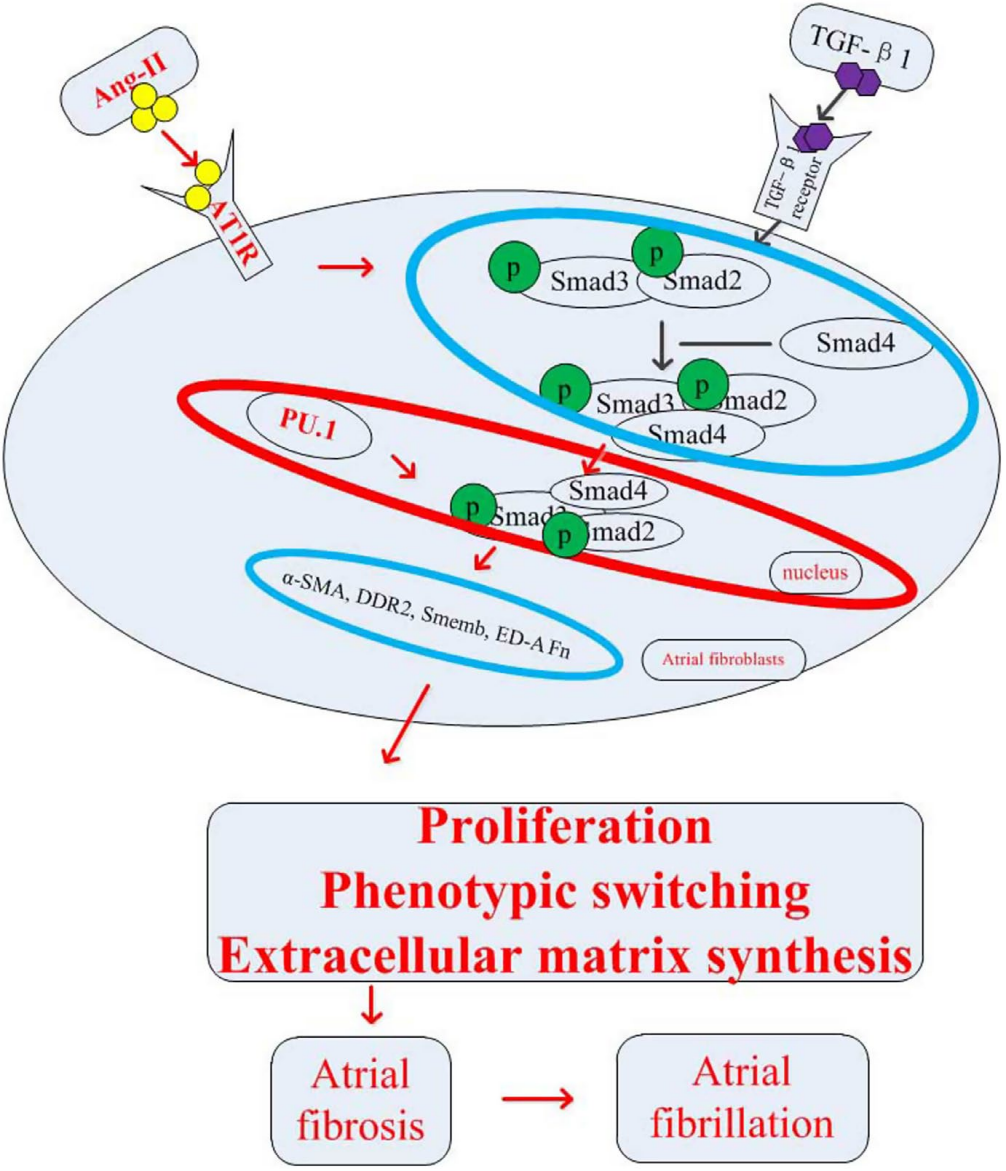
Summary of the roles of PU.1 in atrial fibrosis and vulnerability to atrial fibrillation induced by angiotensin‐II in mice. PU.1 inhibition attenuates atrial fibrosis and vulnerability to atrial fibrillation induced by angiotensin‐II in mice by reducing TGF‐β1/Smads signalling pathway activation. MI: myocardial infarction

To further validate our previous findings suggesting that PU.1 mediates Ang‐II‐induced atrial fibrosis via the TGF‐β1/Smads pathway, because Smad3 is an important regulatory protein of TGF‐β1/Smads pathway,^37^, ^38^ whether PU.1 would affect the activity of TGF/Smads pathway by mediating Smad3. Smad3 expression was knocked down in atrial fibroblasts transfected with a mice PU.1 overexpression (Ad‐PU.1) or control (Ad‐GFP) adenoviral vector. Figure 6A provides representative photomicrographs of the atrial fibroblasts treated with Ad‐GFP or Ad‐PU.1 for 48 h; the results indicate that the fluorescence density is obviously enhanced in two groups. As shown in Figure 6B, immunofluorescence staining indicated that the number of PU.1‐expressing cells was markedly higher in the Ad‐PU.1 group compared with the Ad‐GFP group. Additionally, Western blotting revealed significantly increased (*P* < .01) PU.1 protein expression in the atrial fibroblasts treated with Ad‐PU.1 compared with Ad‐GFP group (Figure 6C, D). Additionally, the siRNA targeting Smad3 significantly reduced (*P* < .01) Smad3 expression compared with the si control based on Western blotting (Figure 6E, F). Further, PU.1 overexpression in Ang‐II‐induced atrial fibroblasts obviously increased proteins of p‐Smad3; on the contrary, Smad3 knock‐down in atrial fibroblasts decreased PU.1 expression (Figure 6G‐J, *P* < .05). In addition, treatment of the atrial fibroblasts with Ang‐II significantly increased the CCK‐8 absorbance values, protein expression of α‐SMA, Smemb and collagen I (*P* < .01), and pre‐treatment with si‐Smad3 attenuated these Ang‐II‐induced effects (*P* < .05); these changes were, in turn, reversed by Ad‐PU.1 treatment (Figure 6K‐O, *P* < .05). These findings suggest that PU.1 inhibition mitigates Ang‐II‐induced atrial fibrosis by at least in part through decreasing TGF‐β1/Smads pathway activation (see Figure 7).

## 4 DISCUSSION

The present study explored the effect of PU.1 on atrial fibrosis and vulnerability to atrial fibrillation (AF). The study findings indicated that PU.1 expression was significantly increased in the mouse and cell models of atrial fibrosis induced by Ang‐II, while PU.1 inhibition reversed these effects. Further, the effect of PU.1 on Ang‐II‐induced atrial fibrosis was at least partially dependent on activation of the TGF‐β1/Smads pathway. These findings suggest that PU.1 is a potential therapeutic target for atrial fibrosis and vulnerability to AF.

In clinical AF, almost 70% of patients present with AF secondary to underlying coronary heart disease, hypertension, rheumatic heart disease, or valvular disease, and in these patients, atrial fibrosis increases AF inducibility and maintains AF.^39^, ^40^, ^41^ We have known that the tissue renin‐angiotensin system (RAS) hyperactivity and high expression are involved in the pathomechanism of cardiac fibrosis^42^; moreover, Ang‐II has also been reported to participate in the maintenance of AF by inducing atrial structural remodelling, which is characterized by interstitial fibrosis.^43^, ^44^ Therefore, blocking the atrial fibrosis induced by Ang‐II may provide a strategy to inhibit the occurrence and maintenance of AF.

PU.1, a transcription factor, is an important regulator of tissue fibrosis.^45^ Earlier studies have shown that PU.1 is expressed in myeloid cells, B cells and so on.^12^, ^13^ Recent research suggests that PU.1 is also highly expressed in fibrotic fibroblasts isolated from liver, lung, and kidney tissue and fibrotic joints; interestingly, resting fibroblasts and inflammatory fibroblasts have not been found to exhibit PU.1 expression.^26^ However, there remains limited information on the expression of PU.1 in Ang‐II‐mediated atrial fibrosis. Therefore, in the present study, PU.1 expression was analysed both in vivo (in mice subcutaneously infused with Ang‐II) and in vitro (in atrial fibroblasts isolated from Ang‐II‐induced mice). The PU.1 inhibitor, DB1976, was used to assess the effect of PU.1 inhibition on Ang‐II‐mediated atrial fibrosis. Treatment with DB1976 in the mice model of subcutaneously infused with Ang‐II did not affect bodyweight and pain (data not shown); it is similar to previous literature reports.^26^ Research has reported that DB1976 do not bind PU.1 but strongly inhibit the PU.1/DNA complex in vitro and fully antagonized PU.1‐dependent transactivation in vivo; importantly, it has minimal effects on other ETS transcription factors.^46^ While PU.1 exhibited low expression in the sham group, subcutaneous Ang‐II infusion significantly up‐regulated PU.1 expression. Similar results were found in vitro. These findings suggest that PU.1 is a key factor in the pathogenesis of atrial fibrosis mediated by Ang‐II.

TGF‐β1 administration has been shown to exert fibrotic effects by enhancing collagen synthesis in vitro and in vivo, with higher levels of TGF‐β1 being associated with more extensive atrial fibrosis.^47^ Moreover, infusion with Ang‐II is reported to result in atrial fibrosis, increased AF inducibility and increased AF duration.^48^ Thus, Ang‐II and TGF‐β1 activation are deemed to play an important role in both atrial fibrosis and electric remodelling.^7^, ^49^ Additionally, Ang‐II exerts its effects by increasing TGF‐β1 expression, as Ang‐II is unable to induce cardiac fibrosis in the absence of TGF‐β1.^1^ Therefore, reducing activation of the TGF‐β1/Smads pathway is expected to abrogate the atrial fibrosis induced by Ang‐II. PU.1, a vital transcription factor, participates in the TGF‐β1/Smad3 pathway to promote collagen expression.^26^ However, whether PU.1 impacts Ang‐II‐mediated TGF‐β1/Smads pathway activation in models of atrial fibrosis was previously unclear. Consistent with an earlier study,^26^ the present study showed that Ang‐II significantly increases the protein expression of TGF‐β1, p‐Smad3 and p‐Smad2/3, and these effects are attenuated by the PU.1 inhibitor, DB1976. This suggests a key role for PU.1 in the TGF‐β1/Smads pathway during the pathogenesis of atrial fibrosis induced by Ang‐II.

Previous studies have demonstrated that atrial fibroblast proliferation and differentiation are vital triggers of cardiac fibrosis. Ang‐II and TGF‐β1 are the most potent stimulators of collagen synthesis in cardiac fibroblasts.^50^, ^51^ We have known that fibroblast proliferation and differentiation into the myofibroblast phenotype contributes to the accumulation of extracellular matrix (including α‐SMA, chemokines, cytokines and adhesion complexes) in the perivascular space and the perimysium surrounding the cardiac muscle bundles.^50^, ^52^, ^53^, ^54^ The recent report has showed that PU.1 can prompt the conversion of fibroblasts into myofibroblasts in tissues of the lung, liver, kidney and skin via the TGF‐β1/Smads pathway.^26^ However, whether PU.1 participates in atrial fibroblast proliferation and differentiation was previously unclear. Especially, evidence shows that cardiac myofibroblasts up‐regulate protein expression of α‐SMA, SMemb and ED‐A Fn; especially, ED‐A Fn is the major driver of myofibroblast phenoconversion, and SMemb and α‐SMA are the two major contractile proteins, which generate tension by contracting to generate force.^55^, ^56^, ^57^, ^58^ Additionally, DDR2 predominantly expresses in fibroblasts and is a fibroblast‐selective marker,^59^ DDR2 has been reported to regulate cell proliferation,^60^ differentiation^61^, ^62^ and extracellular matrix remodelling.^63^ Our results showed that PU.1 inhibition with DB1976 or si‐PU.1 effectively reduced the number of atrial fibroblasts and decreased the expression of PCNA, α‐SMA, SMemb and ED‐A fibronectin and DDR2 in vivo and in vitro. These findings further demonstrate the key role of PU.1 in Ang‐II‐induced atrial fibrosis.

TGF‐β1‐mediated aberrant collagen I is produced by myofibroblasts and is a major constituent of the cardiac interstitium; moreover, TGF‐β1 itself is activated by Ang‐II and TGF‐β1 stimulates CTGF expression.^64^ A large number of studies have shown that PU.1 is implicated in the pathogenesis of fibrosis. For example, inhibition of PU.1 has been shown to ameliorate metabolic dysfunction and non‐alcoholic hepatitis by reducing fibrosis,^25^ PU.1 has been associated with hepatitis C virus–mediated induction of inflammatory responses and fibrosis ^65^ and promotes hepatic fibrosis via activation of the hepatocytes.^28^ Further, PU.1 affects fibrosis of the ocular tissue.^27^ Our results also indicated that the percentage of atrial fibrosis area in vivo was significantly decreased in the DB1976‐exposed group compared with the Ang‐II group based on Masson trichrome staining. In addition, DB1976 was observed to markedly reduce the up‐regulation of CTGF and collagen I induced by Ang‐II. Consistent with these in vivo results, cultured atrial fibroblasts derived from Ang‐II‐induced mice showed significantly reduced CTGF and collagen I expression following treatment with DB1976 or si‐PU.1. It may be deduced that PU.1 inhibition attenuates the atrial fibrosis induced by Ang‐II, which may also lessen vulnerability to AF.

Interstitial fibrosis accelerates conduction, and the presence of interstitial collagen is thought to represent both persistent AF and more rapid longitudinal conduction in patients with AF.^66^, ^67^, ^68^ Additionally, fibroblast proliferation, differentiation and fibrosis in the myocardial interstitium promote the occurrence and maintenance of AF via electrical conduction disturbances.^69^ These disturbances result in anisotropy and re‐entry, eventually leading to the proliferation of ectopic foci and the propagation of irregular wave fronts.^68^, ^70^, ^71^ A recent report demonstrated that the relationship between fibrosis and PU.1‐related effects on the TGF‐β1/Smads pathway exists in the skin, lung, joints, liver and kidney.^26^ Here, we explored whether PU.1 inhibition reduces Ang‐II‐induced atrial fibrosis to further decrease vulnerability to AF. The present study demonstrated that PU.1 inhibition did indeed attenuate AF vulnerability and AF duration in mice post‐Ang‐II infusion. A possible mechanism for this reduction may be that PU.1 inhibition suppresses atrial fibrosis at least in part through blocking the TGF‐β1/Smads pathway.

Evidences show that Smad3 is a primary intracellular signalling molecule along the TGF‐β1/Smads pathway for fibrosis, and down‐regulation of the phosphorylation of Smad3 has been shown to block the pathological process of fibrosis.^72^ Similarly, our goal was also to observe whether PU.1 would affect the activity of TGF‐β1/Smads pathway by mediating Smad3. It is generally accepted that TGF‐β1 binding of ALK5 activates an intrinsic serine/threonine kinase that mainly phosphorylates Smad3, and disruption of Smad3 appears to impair myofibroblast differentiation and restrain the expression of extracellular matrix.^73^ Further, PU.1 can induce Smad3 activation to promote tissue fibrosis,^26^ suggesting a direct impact of PU.1 on atrial fibrosis via TGF‐β1/Smads. However, the specific mechanisms underlying the effects of PU.1 on atrial fibrosis remained to be further explored. Therefore, the present study employed atrial fibroblasts transfected with siRNA to knock down Smad3. The results showed that PU.1 overexpression in Ang‐II‐induced atrial fibroblasts obviously increased proteins of p‐Smad3; on the contrary, Smad3 knock‐down in atrial fibroblasts decreased PU.1 expression. Additionally, si‐Smad3 attenuated the Ang‐II‐induced atrial fibroblast proliferation **(**the CCK‐8 absorbance values**)** and differentiation **(**α‐SMA, SMemb and collagen I**)**, whereas these effects were reversed by the PU.1‐overexpression adenoviral vector, Ad‐PU.1. These findings indicate that PU.1 inhibition attenuates the vulnerability to AF and AF duration induced by Ang‐II, and the mechanisms underlying these effects may be related to the reduced activation of TGF‐β1/Smads pathway.

In conclusion, PU.1 inhibition can attenuate Ang‐II‐induced atrial fibrosis and consequently decrease vulnerability to AF by suppressing activation of the TGF‐β1/Smads pathway. These findings suggest that PU.1 may be a promising therapeutic target for the treatment of atrial fibrosis and the reduction of AF vulnerability.

## CONFLICT OF INTEREST

The authors have no conflicts of interest to declare.

## AUTHOR CONTRIBUTION

**Juan Hu:** Conceptualization (equal); Data curation (equal); Formal analysis (equal); Funding acquisition (equal); Investigation (equal); Methodology (equal); Project administration (equal). **Jing Jing Zhang:** Conceptualization (equal); Data curation (equal). **Li Li:** Investigation (equal); Methodology (equal). **Ling Shan Wang:** Software (equal); Validation (equal). **Tao Hai Yang:** Methodology (equal); Visualization (equal). **Wei Xian Fan:** Software (equal); Supervision (equal). **Ming Lei Zhang:** Investigation (equal); Project administration (equal). **Ling Guang Hu:** Funding acquisition (equal); Investigation (equal); Writing‐review & editing (equal). **Xia Hai Fu:** Supervision (equal); Validation (equal). **Feng Wei Song:** Methodology (equal); Project administration (equal). **Jie Li Yan:** Software (equal); Supervision (equal). **Jing Jing Liu:** Methodology (equal); Visualization (equal). **Tao Jin Wu:** Data curation (equal); Formal analysis (equal); Investigation (equal). **Bin Kong** **:** Data curation (equal); Visualization (equal); Writing‐original draft (equal).
